# Risk Overgeneralization in Times of a Contagious Disease Threat

**DOI:** 10.3389/fpsyg.2020.01392

**Published:** 2020-06-16

**Authors:** Spike W. S. Lee, Julie Y. Huang, Norbert Schwarz

**Affiliations:** ^1^Rotman School of Management, Department of Psychology, University of Toronto, Toronto, ON, Canada; ^2^College of Business, Stony Brook University, Stony Brook, NY, United States; ^3^USC Dornsife Mind and Society Center, USC Dornsife Department of Psychology, USC Marshall School of Business, University of Southern California, Los Angeles, CA, United States

**Keywords:** COVID-19, disease threat, feelings, risk perception, policy preference, xenophobia

## Abstract

People’s assessment of risks is swayed by their current feelings. COVID-19 invokes powerful feelings because it is (i) a salient, enormous threat, (ii) unfamiliar, and (iii) intertwined with xenophobia. These three factors are known to exert predictable influence on people’s risk overgeneralization, policy preference, and sociopolitical attitudes. We provide a succinct, illustrative review of empirical work on these dynamics in times of a disease outbreak (e.g., the 2009 H1N1 swine flu, the 2014 Ebola). Theoretical and applied implications for the present COVID-19 pandemic include the value of salience in motivating public opinion change, the importance of reducing unfamiliarity for curbing risk-averse tendencies, and the need for policies that guard against xenophobia-driven racism in collaborative efforts.

## Introduction

When people assess risks, they use not only facts and data, but also their current feelings (Loewenstein et al., 2001). As emotions run high in times of a contagious disease threat, people tend to perceive higher health risks and overgeneralize them, which exerts predictable influence on sociopolitical attitudes. COVID-19 provides a powerful illustration of this phenomenon because it is (i) a salient, enormous threat, (ii) unfamiliar, and (iii) intertwined with xenophobia. In this brief, we provide an illustrative review of primarily experimental and some correlational research on the psychology of health risks, particularly behavioral insights gained from prior outbreaks (e.g., 2009 H1N1, 2014 Ebola), and suggest ways in which they shed light on specific psychological reactions to the present COVID-19 pandemic.

## A Salient, Enormous Threat

“Dr. Neil M. Ferguson, a British epidemiologist who is regarded as one of the best disease modelers in the world, produced a sophisticated model with a worst case of 2.2 million deaths in the United States.” (Kristof, 2020)

In the face of a salient, enormous threat to humanity, fear knows no limits. Consider research findings from the 2009 H1N1 (“swine flu”) pandemic. Within a matter of 2 months, the World Health Organization declared a public health emergency of international concern as the H1N1 virus spread to more than 70 countries and all 50 states in the United States. Media coverage was extensive, highlighting the risk of contagion and the importance of frequent hand washing and avoiding interpersonal physical contact (CDC, 2019a). What was the impact of such a salient disease threat on people’s risk perception and policy preference?

Two naturalistic experiments illustrate it (Lee et al., 2010). The first experiment was conducted 3 weeks after the first documented case of human infection in the United States, while H1N1 remained the primary focus of media attention. Passers-by were approached on a university campus and asked to estimate “the probability that the average American may experience the following events within the next 12 months: Contracting a serious disease; Having a heart attack before age 50; Dying from crime or accident.” These questions tapped into perception of a health risk directly related to H1N1, a health risk unrelated to H1N1, and a non-health risk. Respondents were also asked to express their overall view of the U.S. health care system by completing an item adapted from a New York Times/CBS News poll.

To test the influence of disease salience on these measures, a confederate would walk by and loudly sneeze and cough (experimental condition) or just walk by without a sneeze or cough (control condition) before respondents answered the questions. Risk estimates were higher by an average of 13.7 percentage points in the experimental condition than in the control condition. The increase was observed regardless of whether the risk was related to H1N1, unrelated to it, or even unrelated to health. Disease salience also led respondents to express a less favorable view of the health care system and to consider it more in need of complete rebuilding.

The second experiment was conducted 3 weeks later and involved a similar sneezing procedure. This time, passers-by in the downtown area of a college town were asked if they preferred spending a $1.3 billion federal investment “(1) to facilitate the production of flu vaccines, or (2) to create green jobs.” Without disease salience, 16.7% of respondents favored flu vaccine production. With disease salience, 47.8% did.

These findings have several implications. The most obvious is that risk perception can be heightened and overgeneralized to unrelated domains, consistent with prior work (Johnson and Tversky, 1983). Risk overgeneralization is likely to contribute to risk-averse behaviors across the board, as exhibited in the present pandemic, from hoarding products at the individual level to market sell-off at the collective level.

The public’s attitude toward the health care system and preference for federal spending (e.g., on vaccine production) can move dramatically in the midst of a palpable crisis. Public opinions on other policies are likely to ride on the wave as well, as in current rising support for paid sick leave for family-care and a direct governmental payout among other institutional changes (Miller, 2020; Murad, 2020).

In motivating these public opinion changes, policymakers want to ensure the disease threat is salient. As the sneezing experiments illustrate, making a health threat salient in the moment exerts an influence even when the threat already has extensive media coverage. Different focal concerns come to mind in the course of everyday life, and salience inductions—even as simple as the use of face masks and the maintenance of six feet apart—ensure attention on the threat and increase its behavioral impact.

## Unfamiliarity

“Novel coronavirus (2019-nCoV)” (World Health Organization [WHO], 2020).

New and unfamiliar threats often feel riskier than old and familiar ones. The feeling is adaptive insofar as caution around unknowns is warranted. “As the late psychologist Robert Zajonc liked to say, ‘If it’s familiar, it hasn’t eaten you yet”’ (Burkeman, 2011, 153), whereas new and unfamiliar threats may. Indeed, feelings of unfamiliarity with an entity can heighten people’s perception of its riskiness (e.g., perceived health hazards of food additives; Song and Schwarz, 2009), resulting in reduced investment in stocks (Alter and Oppenheimer, 2006; Green and Jame, 2013) and reduced trust in others (Silva et al., 2017).

Coronavirus has “novel” right in its name. It was first found in a Chinese city many people had never heard of. Facts about it—infection and mortality rates, geographical reach and time course of spread, treatment options, long-term effects—were unfamiliar or unknown. All of these reinforce perception of its riskiness. As the pandemic unfolds, however, facts will emerge. Insights will be gained. Information will become available. Transparency is key. Delay or suppression of information undermines public trust and sustains public fear, as was evident in China, where the government’s handling of information about coronavirus caused a wave of widespread, publicly expressed skepticism about the government’s intentions and competence (Yuan, 2020), which is rare under the Communist Party’s rule. In more open, democratic societies, it is crucial that the public be continuously informed, without delay, in ways that they can comprehend (e.g., by using properly designed visual aids; Garcia-Retamero and Cokely, 2013). It seems obvious, but failing to do so would prolong heightened risk perception and risk-averse behavior due to a sense of unfamiliarity.

## Xenophobia

“A single word scrawled in black marker stood out among the prepared remarks President Trump planned to deliver during Thursday’s White House press briefing on the ongoing global coronavirus pandemic. In the president’s notes, ‘Corona’ had been crossed out and replaced with ‘Chinese’” (Chiu, 2020).

Xenophobia is more than phobia. It refers to “fear and hatred of strangers or foreigners or of anything that is strange or foreign” (Merriam-Webster, 2020). Research on the behavioral immune system—the psychological and behavioral mechanisms that people use to avoid disease (Schaller and Park, 2011; Schaller, 2015) and that both activate and are activated by the biological immune system (Schaller et al., 2010; Miller and Maner, 2011)—suggests that contagious disease threats sensitize people not only to personal risks (as described above), but also to intergroup risks, resulting in increased prejudice against outgroups.

Consider illustrative evidence from the height of the H1N1 pandemic again. An online experiment in the United States during Fall 2009 (Huang et al., 2011) found that participants reported more racist attitudes toward immigrants (e.g., “Over the past few years, immigrants have gotten more economically than they deserve”; McConahay, 1986) if they had previously read a news article regarding swine flu’s health risks and limited vaccine supply than if they had not been given the article to read. This effect, however, only emerged among participants who had not been vaccinated; it did not emerge among participants who had already been vaccinated, nor did it emerge for participants who had not read the article. Converging evidence came from a lab experiment among U.S. undergraduates, which showed that more germ-aversive participants expressed more unfavorable attitudes toward outgroups after reading a news article about the seasonal flu. If they had been given an opportunity to clean their hands with an antiseptic wipe, however, they did not express more unfavorable attitudes.

These findings are consistent with the notion that people’s proclivities toward avoiding disease risk can produce overgeneralized avoidance of foreign, unfamiliar entities (e.g., immigrants and other out-groups) even when they are not actual disease vectors (Schaller and Duncan, 2007). Vaccination and hand hygiene—two protective mechanisms against disease—have the potential to weaken perceived disease risks and subsequently, the corresponding xenophobia.

Similar conclusions can be drawn from correlational research. For example, survey data from a nationally representative American sample during the 2014 Ebola outbreak showed that the more vulnerable to Ebola participants perceived themselves to be, the more they exhibited generalized xenophobia (Kim et al., 2016). Meta-analytic data across 24 studies also found that disease avoidance tendencies (e.g., fear of contamination, disgust sensitivity) are associated with ethnocentrism and other forms of social conservatism motivated by exclusivity and negativity toward outgroups (Terrizzi et al., 2013). Longitudinal analyses of U.S. polling data before and after the Ebola outbreak found that the amount of Internet searches for “Ebola” was associated with increased inclinations to vote for Republican candidates in Senate and House of Representatives elections—only in states with norms that already favored Republican candidates (Beall et al., 2016).

Such dynamics of the behavioral immune system can tear apart or mend the fabrics of a diverse society. They are likely to be particularly powerful in the present pandemic for two reasons. First, coronavirus appears considerably more severe than the 2009 H1N1 and the seasonal flu (CDC, 2019a, 2020a,b). Second, a vaccine is not yet available. Until it is, we must depend on community non-pharmaceutical interventions like physical distancing and temporarily closing public spaces (CDC, 2019b). The success of these interventions, however, may be limited by “us” versus “them” thinking since they require coordination and cooperation of multiple actors, from individuals to governmental institutions (e.g., New York, Connecticut, and New Jersey coordinated their anti-coronavirus restrictions to optimize the effectiveness of these policies; Lovelace et al., 2020). When the success of public health responses depends on all parties comprehending the seriousness of the health risk and jointly complying with behavioral restrictions, illness-ignited xenophobia and prejudices constitute a particularly insidious social threat.

Consequently, it behooves governments and organizations to stay mindful of the link between disease risk and xenophobia, even as they seek ways to keep the public’s attention on coronavirus. To that end, the WHO explicitly cautions against naming or referring to human diseases by culture or geography (World Health Organization [WHO], 2015). A media advocacy group is urging fellow journalists to avoid using images of East Asians in face masks and U.S. Chinatowns when describing coronavirus (Asian American Journalists Association [AAJA], 2020). Deviating from official nomenclature, President Trump has been calling coronavirus the “Chinese virus,” directing blame to the outgroup and away from the government’s response, while fueling anti-Chinese and anti-Asian racism and hate crimes (Carlisle, 2020; Tavernise and Oppel, 2020).

These dynamics make actions such as border-closing particularly appealing, which is likely to reinforce the division between “us” and “them,” wherever the line is drawn (e.g., Americans vs. non-Americans, Caucasians vs. Asians, New Yorkers vs. Floridians). But when coronavirus is already spreading through local communities, and race/ethnicity fails to provide valid predictors of who is or is not a carrier, the false sense of security and psychological satisfaction provided by such policies can backfire, especially at a time when massive collaborative efforts are needed.

## Summary

COVID-19 is a salient, enormous threat that cuts across national and racial/ethnic boundaries. As a contagious disease threat, many facts about it remain unfamiliar to most ordinary people, and its global origins predispose its spread to worsen xenophobia. Consequences such as risk overgeneralization and risk-averse behaviors are hard to curb unless and until trustworthy information and effective prevention and treatment become available, which will reduce the enormity and unfamiliarity of the threat.

By highlighting the consequences of powerful feelings invoked by COVID-19 throughout this article, we hope to convey pros and cons for practitioners and policymakers to consider: On the one hand, they have to emphasize the magnitude of the population-level risk from coronavirus and how urgent action by everyone is needed. On the other hand, the same urgency they wish to communicate may drive up risk-averse behaviors, xenophobic attitudes, and potential harm against already marginalized populations. Concrete solutions and guidelines for reducing these undesirable consequences should be in place alongside other public health recommendations.

## Data Availability Statement

The original contributions presented in the study are included in the article/supplementary material; further inquiries can be directed to the corresponding author/s.

## Author Contributions

All authors listed have made a direct intellectual contribution to the work and approve it for publication.

## Conflict of Interest

The authors declare that the research was conducted in the absence of any commercial or financial relationships that could be construed as a potential conflict of interest.
